# Modulation of cyclins and p53 in mesangial cell proliferation and apoptosis during Habu nephritis

**DOI:** 10.1007/s10157-015-1163-6

**Published:** 2015-09-10

**Authors:** Yang Lu, Jun Wen, DaPeng Chen, LingLing Wu, QingGang Li, Yuansheng Xie, Di Wu, Xiaoluan Liu, XiangMei Chen

**Affiliations:** Department of Nephrology, Chinese PLA General Hospital, Chinese PLA Institute of Nephrology, Beijing Key Laboratory of Kidney Disease, State Key Laboratory of Kidney Diseases, National Clinical Research Center for Kidney Diseases, Fuxing Road 28, Beijing, 100853 People’s Republic of China; Department of Nephrology, China-Japan Friendship Hospital, Beijing, People’s Republic of China

**Keywords:** Cell cycle, Mesangial proliferative nephritis, Cyclins, p53

## Abstract

**Background:**

Mesangial cell (MC) proliferation and apoptosis are the main pathological changes observed in mesangial proliferative nephritis. In this study, we explored the role of cyclins and p53 in modulating MC proliferation and apoptosis in a mouse model of Habu nephritis.

**Methods:**

The Habu nephritis group was prepared by injection of Habu toxin. Mesangiolysis and mesangial expansion were determined by periodic acid-Schiff (PAS) reagent staining. Immunohistochemical analysis of PCNA and KI67, and TUNEL staining were used to detect cell proliferation and apoptosis, respectively. Expression levels of cyclins and p53 were examined by Western blotting.

**Results:**

PAS staining showed that mesangial dissolution appeared on days 1 and 3, and mesangial proliferation with extracellular matrix accumulation was apparent by days 7 and 14. Both PCNA and KI67 immunohistochemical analysis showed that MC proliferation began on day 3, peaked on day 3 and 7, and recovered by day 14. TUNEL staining results showed that MC apoptosis began to increase on day 1, continued to rise on day 7, and peaked on day 14. Western blot analysis showed that cyclin D1 was upregulated on day 1, cyclins A2 and E were upregulated on days 3 and 7, and p53 was upregulated on days 3, 7 and 14. There was no change in the expression levels of Bax or p21.

**Conclusion:**

We explored the tendency for MC proliferation and apoptosis during the process of Habu nephritis and found that cyclins and p53 may modulate the disease pathology. This will help us determine the molecular pathogenesis of MC proliferation and provide new targets for disease intervention.

## Introduction

Mesangial proliferative glomerulonephritis (MesPGN), including IgA nephropathy, lupus nephritis and purpura nephritis, is a common clinical glomerulonephritis and the main cause of end-stage renal disease in China [1]. The main feature of MesPGN is abnormal mesangial cell (MC) proliferation with increased extracellular matrix expression and progressive glomerular scarring [2]. Habu snake venom glomerulonephritis is a well-established model of MesPGN in mice with self-limited glomerular injury. It includes three important pathological processes. The first is the mesangiolysis phase, with mesangial dissolution from days 1 to 3 after tail vein injection with Habu venom. In the second phase, from days 3 to 7, MC proliferation promotes the repair of mesangial dissolution, involving many MCs and extracellular matrix (ECM) filling nearly the entire cyst [3]. In the recovery phase (days 14–21), following clearance of the stimulus by the immune system, overgrowing MCs undergo apoptosis, the ECM is absorbed, and the injured glomerulus gradually recovers its normal structure. Throughout the process of Habu nephritis, activation of cell proliferation and apoptosis accompany each other to mediate the development and recovery of mesangial proliferation. Hartner et al. [4] showed that both activation of cell proliferation and apoptosis peaked at day 7 in Habu nephritis with unilateral nephrectomy. However, previous studies did not provide the precise proliferative trend, via detection of molecular markers such as PCNA or KI67, or the overall apoptosis trend during the process of Habu nephritis.

Many molecular mechanisms involving cyclins (cyclin A, p27), growth factors (PDGF, IGF-1, EGF, HGF, etc.), vasoactive substances (ET-1, ANGII), cytokines (IL-6, IL-10, IL-12), transcription factors (YB-1, FHL2), and matrix metalloproteinases are involved in the pathogenesis of mesangial proliferative glomerulonephritis [4–11]. Among these disease-related proteins, cyclins and p53 signaling pathways play a direct role in MC proliferation and apoptosis [12]. For example, cyclin D promotes quiescent cells in the G0 phase to enter the early G1 phase, cyclins E and A are essential for the G1/S transition [13], while cell cycle-inhibiting proteins, such as p53, p21 and Bax, can lead to cell cycle arrest or apoptosis [14, 15]. However, the expression patterns of cyclins and p53 in Habu nephritis are unknown.

Therefore, in this study, we examined changes in MC proliferative and apoptotic statuses by detecting molecular markers and explored the expression patterns of cyclins and p53, which will help us understand the mechanisms of pathogenesis in mouse mesangial proliferative nephritis.

## Materials and methods

### Animal model

A total of 80 specific pathogen-free male C57BL/6 mice (18–20 g) were purchased from Aberford Experimental Animal Technology Co., Ltd. (Beijing, China) and housed in cages at constant room temperature (20 °C) and humidity (70 %) under a controlled light–dark cycle in the Experimental Animal Center of the PLA General Hospital. The Habu snake venom glomerulonephritis mouse model was induced using a single intravenous injection of HSV (trimeresurus flavoviridis; 2.5 mg/kg body weight; Wako Pure Chemical Industries, Osaka, Japan) through the tail vein. Control mice (*n* = 16) were injected with an equal volume of normal saline and killed on day 0. Habu mice were killed on days 1, 3, 7, and 14 after injection (16 mice per time point). Glomeruli were isolated by the differential sieving method. Briefly, the renal cortex was minced with scissors and filtered through 140, 75 and 53-μm sieves. Glomeruli obtained using the 53-μm sieve were harvested, with approximately 80 % purity. The glomeruli from four mice were pooled into one sample.

Blood samples were collected by cardiac puncture, and a 24-h urine sample (four mice were pooled into one sample) was collected in metabolic cages. Renal function was evaluated using serum creatinine and blood urea nitrogen levels and the urine albumin/creatinine ratio.

Animal welfare and experimental procedures were carried out strictly in accordance with the Guide for the Care and Use of Laboratory Animals (National Research Council of USA, 1996).

### Renal histology

Kidney tissues were fixed in a 10 % formaldehyde solution and dehydrated using a graded ethanol series. Tissues were embedded in paraffin and cut into 4-μm thick sections. Sections were stained with periodic acid-Schiff (PAS) reagent and counterstained with hematoxylin. The numbers of cells in 30 glomeruli per mouse were counted to quantify the degree of cell proliferation.

### Immunohistochemistry

We used PCNA and KI67 immunohistochemistry to detect cell proliferation. Paraffin sections (4 μm) were mounted onto poly-l-lysine-coated slides, deparaffinized in xylene and rehydrated in alcohol. To block endogenous peroxidase activity, renal sections were reacted with 3 % hydrogen peroxide for 20 min at room temperature. Sections in sodium citrate buffer (pH 6.0) were then heated in a microwave oven for 10 min. After blocking with 1.5 % normal goat serum for 20 min, sections were incubated with a mouse polyclonal antibody against PCNA antigen (1:500, Cell Signaling Technology, USA) and a rabbit polyclonal KI67 antigen (1:200, Abcam, USA) at 4 °C overnight. Sections were then reacted with a biotinylated secondary antibody at room temperature for 45 min after removing the unbound primary antibody and rinsing with phosphate-buffered saline (PBS). Then, the sections were reacted with horseradish peroxidase (HRP)-streptavidin working buffer (SP-9002 HistostainPlus Kits, ZYMED, USA) for 45 min at room temperature. The Vecta-stain DAB Kit (Vector Lab, USA) was used as the chromogen. Finally, each slide was counterstained with hematoxylin. For negative controls, the primary antibody was replaced with PBS. The PCNA and KI67 positive rates were calculated as the number of positive cells relative to that of total glomerular cells.

### TUNEL assay

We performed a terminal deoxynucleotidyl transferase-mediated deoxyuridine triphosphate nick end labeling (TUNEL) assay according to the manufacturer’s instructions (Merck Millipore, Billerica, MA, USA). TUNEL-positive cells, which were stained brown, were counted under 400× magnification. We calculated the percentage of TUNEL-positive cells relative to the total number of glomerular cells as the apoptotic rate.

### Western blot analysis

Mice glomeruli were lysed in RIPA buffer containing a protease inhibitor cocktail (1 μg/ml leupeptin, 1 μg/ml aprotinin, 100 μM PMSF) and centrifuged (30 min, 4 °C, 15,000 rpm). Protein concentration of the supernatant was measured using a BCA Protein Assay Kit (Thermo Fisher Scientific, USA). Protein samples (80 μg) were boiled for 5 min at 95 °C in reducing 1× SDS buffer and subjected to 12 % sodium dodecyl sulfate polyacrylamide gel electrophoresis (SDS-PAGE). Proteins were electroblotted onto nitrocellulose membranes, blocked with blocking buffer for 1 h at room temperature and incubated overnight at 4 °C with primary antibodies against cyclin D1, cyclin A2, p53 (Proteintech Group, USA), cyclin E, Bax, β-actin (Abcam, USA), and p21 (Novus Biologicals, USA). Before and after incubation with secondary antibodies for 1.5 h, the membranes were washed with Tris-buffered saline containing Tween 20 (TBST) buffer three times at room temperature. Enhanced chemiluminescence followed by exposure to X-ray film was used to detect the bound antibodies.

### Statistical analysis

Statistical analyses were performed using SPSS version 17.0. Data are expressed as the mean ± standard deviation (SD). Statistical significance (defined as *p* < 0.05) was evaluated by one-way analysis of variance (ANOVA).

## Results

### Mesangiolysis and MC proliferation with mesangial expansion were the major pathological changes during Habu nephritis

**Fig. 1:**
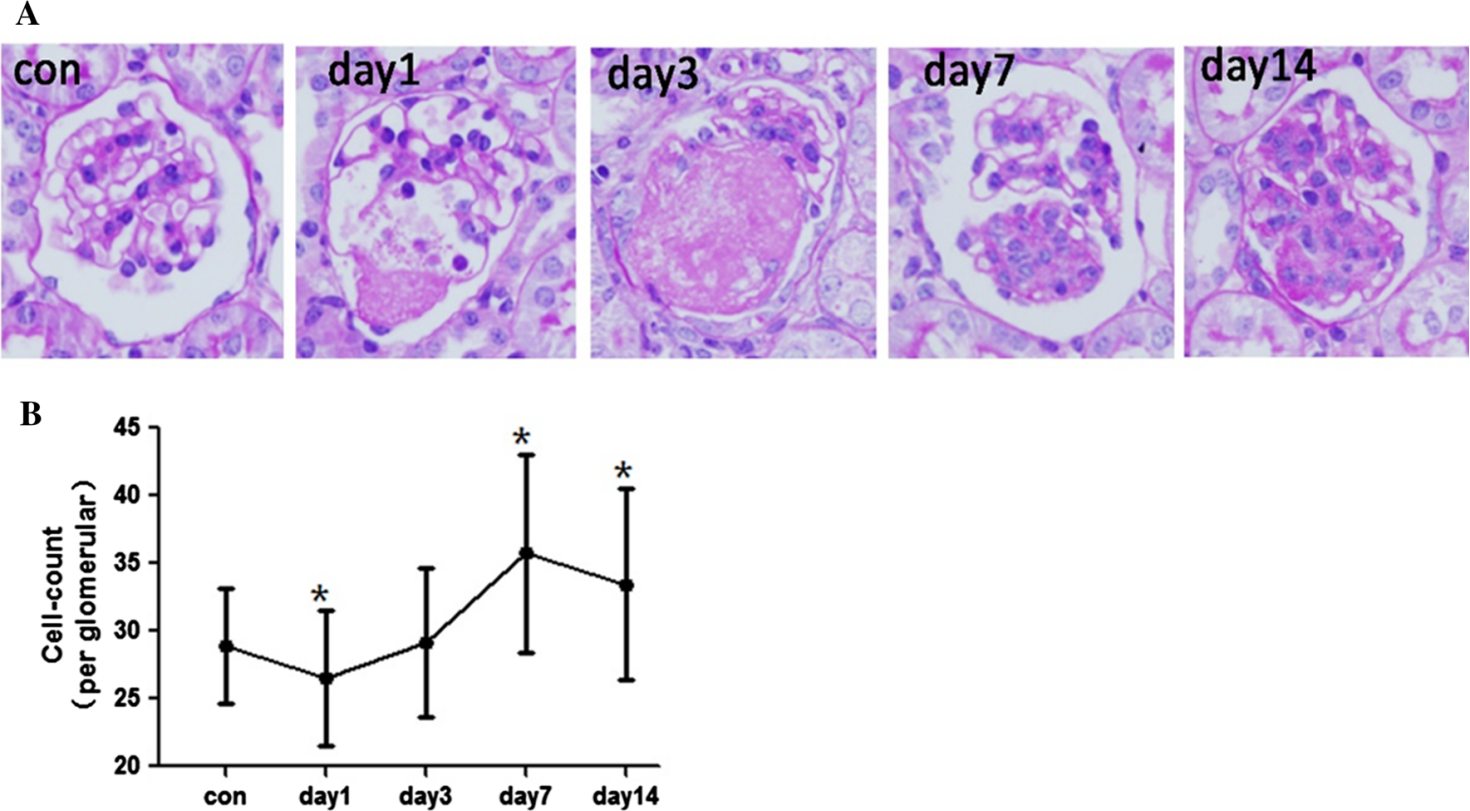
Mesangiolysis and MC proliferation with mesangial expansion were the major pathological changes during Habu nephritis. **a** PAS staining showed that mesangial dissolution appeared on days 1 and 3 with fibrinoid material deposition, and mesangial proliferation with extracellular matrix accumulation was apparent by days 7 and 14 with extracellular matrix accumulation. Control mice were injected with an equal volume of normal saline and killed at day 0 (Con group). **b** The glomerulus cell count results showed that cell number decreased on day 1, than began to increase and peaked on day 7, and recovered on 14, four mice per group, data are mean ± SD; *represents *P* < 0.05 compared to Con

We used PAS staining in combination with glomerulus cell count to examine the pathological changes during Habu nephritis (Fig. 1a, b). There were two major pathological changes: mesangiolysis and mesangial proliferation. The mesangial dissolution appeared on day 1 with decreased mesangial cells, peaked on day 3 with fibrinoid material deposition, and returned to normal by day 7. Mesangial proliferation with ECM accumulation was apparent by day 7 and peaked on day 14.

**Fig. 2:**
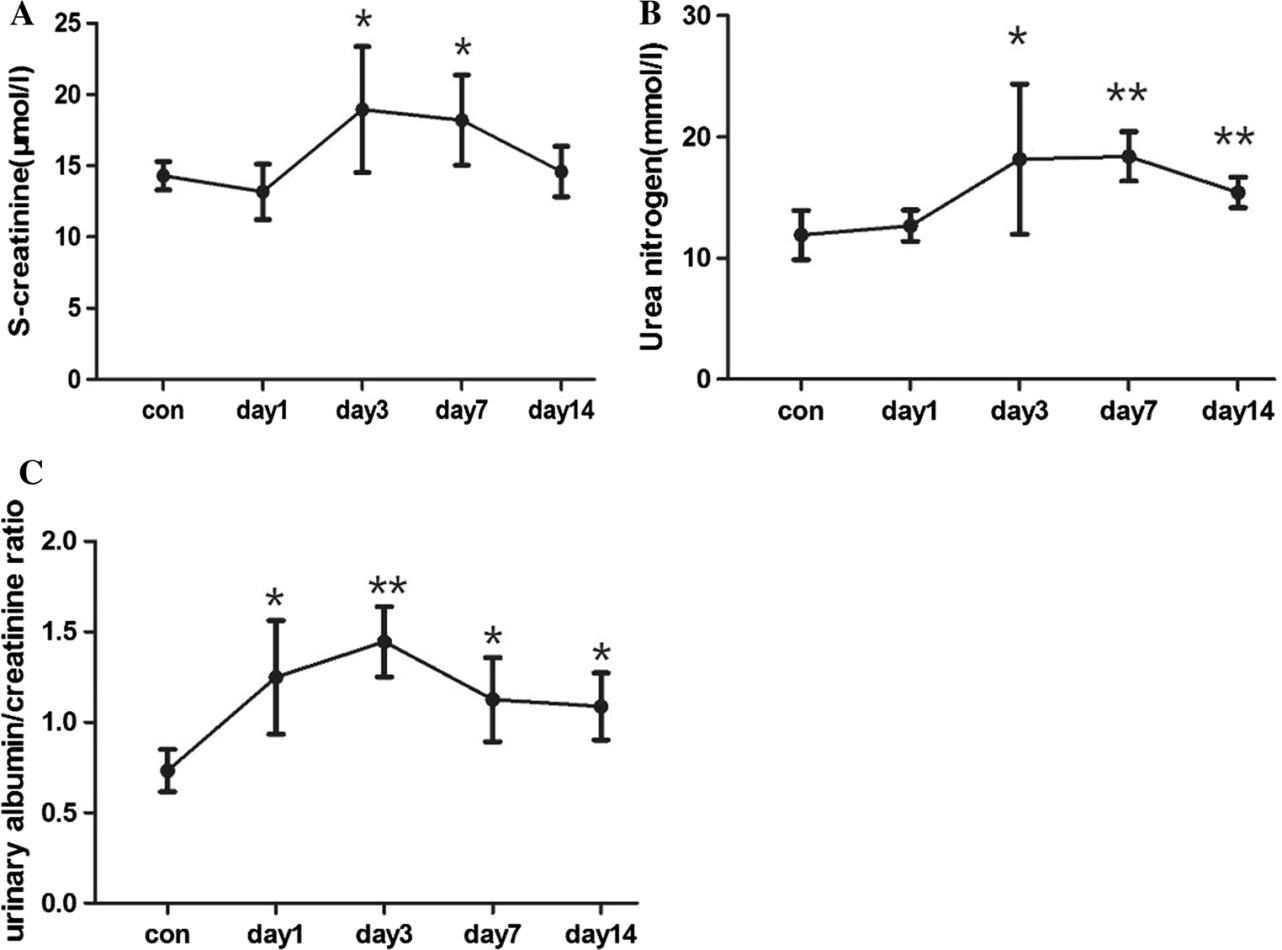
Renal function damage by examining serum creatinine and blood urea nitrogen levels and the urine albumin/creatinine ratio. The Serum creatinine (**a**) and blood urea nitrogen (**b**) increased on days 3 and 7 but decreased by day 14. six mice per group,data are mean ± SD, *represents *P* < 0.05 compared to Con. C. The urine albumin/creatinine ratio increased rapidly on day 1, peaked on day 3 and decreased by day 7, four samples per group (four mice samples pooled for each), data are mean ± SD, *represents *P* < 0.05 compared to Con, **represents *P* < 0.01 compared to Con

We determined renal function damage by examining serum creatinine and blood urea nitrogen levels and the urine albumin/creatinine ratio. Serum creatinine and blood urea nitrogen increased on days 3 and 7 but decreased by day 14 (Fig. 2a, b). The urine albumin/creatinine ratio increased rapidly on day 1, peaked on day 3, and decreased by day 7 (Fig. 2c). We deduced that the rise in the urine albumin/creatinine ratio was associated with glomerular barrier damage in mesangiolysis, while serum creatinine and blood urea nitrogen were related to the decreased glomerular filtration rate in the mesangial proliferative phase.

### The regulatory mechanism of cell proliferation and apoptosis in the pathogenesis of Habu nephritis

**Fig. 3:**
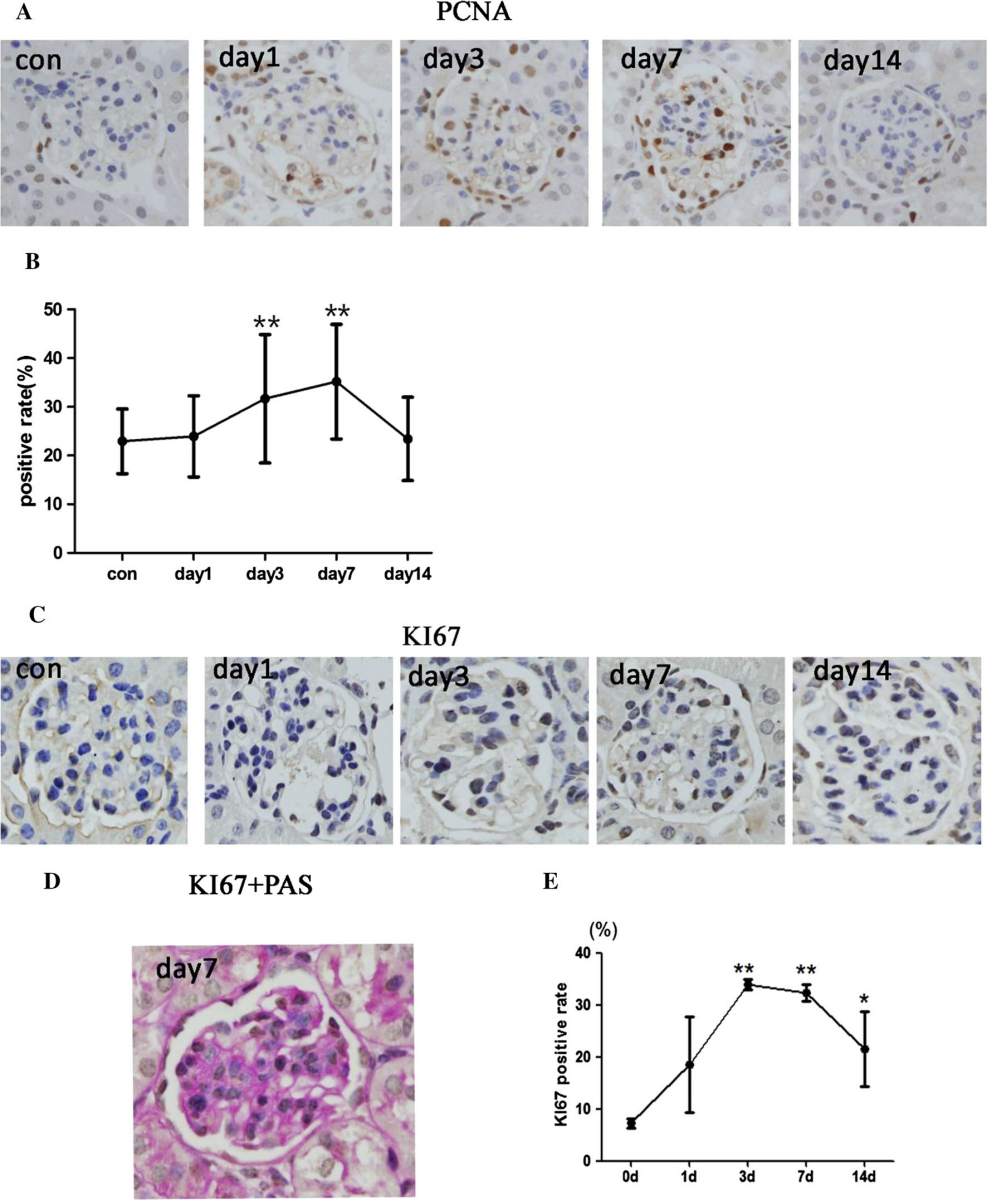
Mesangial cell proliferation was detected by PCNA and KI67 immunohistochemistry. **a** Immunohistochemistry graph of PCNA. **b** PCNA positive rates (mean ± SD) in each group, *n* = 3 mice per group, (10–15 glomeruli detected for each mice). The rate increased on day 1, peaked by days 3 and 7, and subsequently decreased at day 14. **c** Immunohistochemistry graph of KI67. **d** Double staining of KI67 immunohistochemistry with PAS. **e** KI67 positive rates (mean ± SD) in each group. The trend of KI67 was similar to PCNA results. Four mice per group, *represents *P* < 0.05 compared to Con, **represents *P* < 0.01 compared to Con

**Fig. 4:**
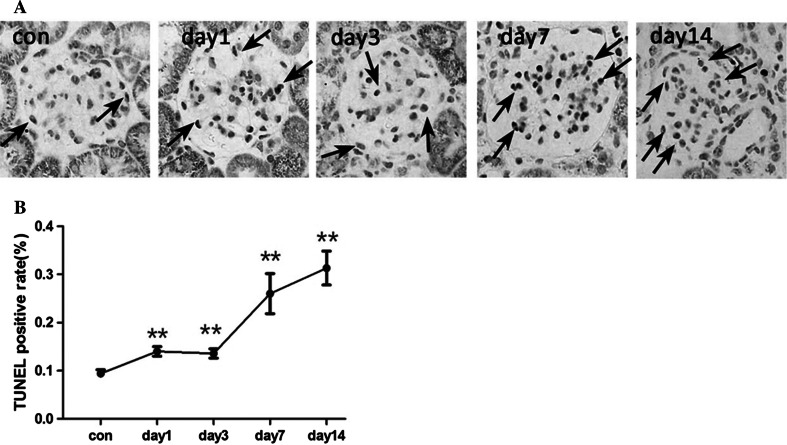
Mesangial cell apoptosis was detected by TUNEL staining. A TUNEL staining graph. B. Apoptosis rates calculated by the percentage of TUNEL-positive cells relative to the total number of glomerulus cells. The apoptosis rate increased from days 1 to 7 and peaked on day 14, three mice per group (30 glomeruli detected for each mice),data are mean ± SD, *represents *P* < 0.05 compared to Con, **represents *P* < 0.01 compared to Con

We detected MC apoptosis using the TUNEL assay and MC proliferation using PCNA and KI67 immunohistochemistry. Both the PCNA and KI67 results showed that the cell proliferation rate began to increase on day 1, peaked on day 3 and 7, and recovered on day 14 (Fig. 3). Moreover, we performed double staining of KI67 immunohistochemistry and PAS and found that most of proliferative cells were MCs in mesangial area. The TUNEL assay results showed that the MC apoptotic rate increased from days 1 to 7 and peaked on day 14 (Fig. 4). These results show that MC proliferation and apoptosis play a crucial role in mediating the pathological process of Habu nephritis.

### Cyclin expression patterns and p53 signaling pathways in Habu nephritis

**Fig. 5:**
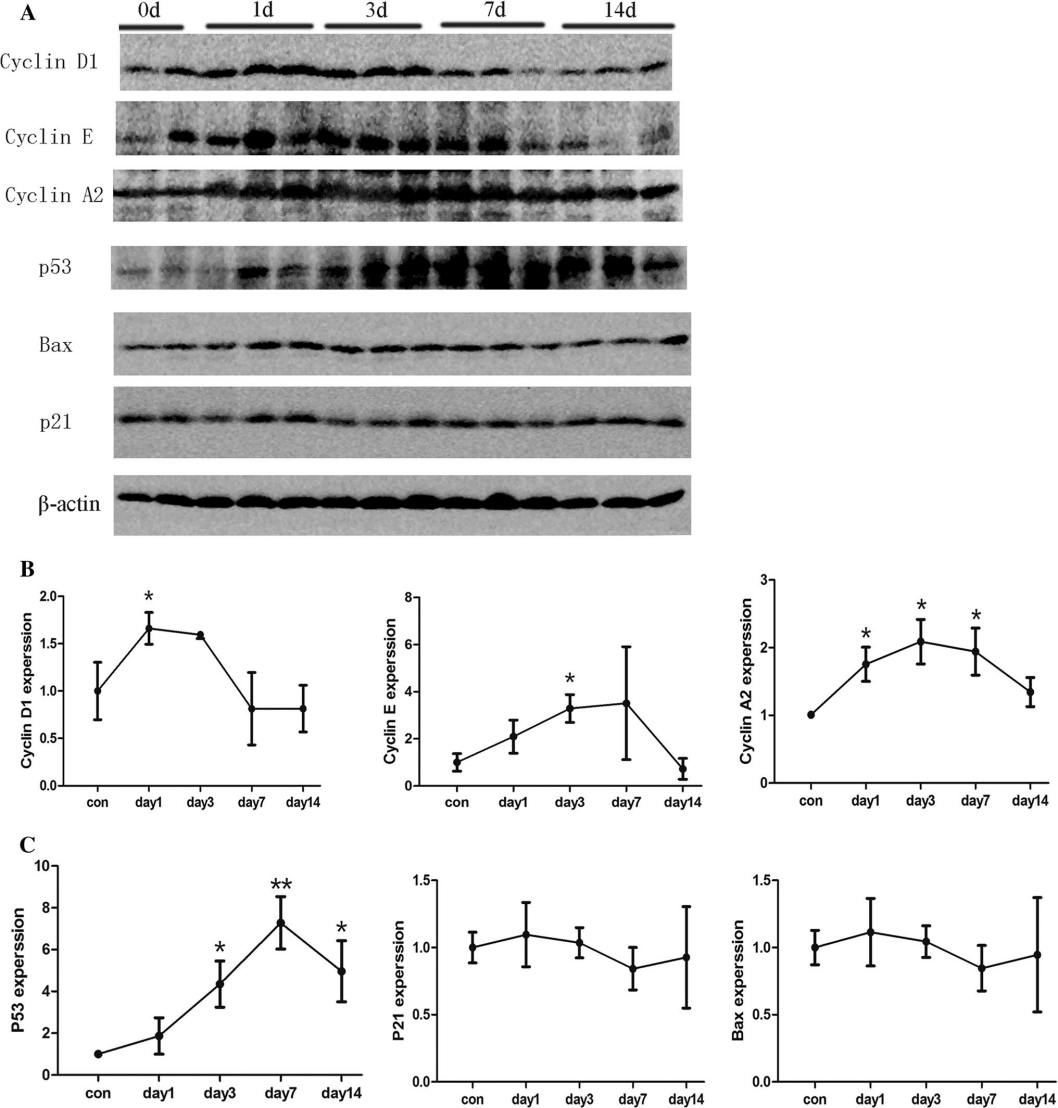
The expression of cell cycle regulatory proteins in the glomeruli of Habu nephritis was examined by Western blotting. **a** Positive cell cycle regulatory proteins including cylins A, D1 and E, and negative regulatory proteins including p53, p21 and Bax were examined by Western blotting. **b** The expression trends of positive cell cycle regulatory proteins in Habu nephritis. Cyclin D1 was mainly up-regulated during the mesangiolysis phase (days 1 and 3), cyclins A and E were upregulated on day 3, peaked on day 7, normal by day 14. **c** The expression trends of negative cell cycle regulatory proteins in Habu nephritis. p53 increased from days 1 to 7 and peaked on days 7 and 14. However, there was no change in the expressions of Bax or p21 during Habu nephritis (the intensities of these protein bands were presented as their ratios to theβ-actin levels and data from the control group were arbitrarily set as 1.0). Data are mean ± SD, *represents *P* < 0.05 compared to Con, **represents *P* < 0.01 compared to Con

We monitored the expression of cell cycle regulatory proteins during Habu nephritis (Fig. 5a). Cyclins A, D1 and E are positive cell cycle regulatory proteins. Cyclin D1 was mainly upregulated during the mesangiolysis phase (days 1 and 3), which is associated with activation of MC proliferation (Fig. 5b). The expression trends of cyclins A and E were similar to the PCNA staining pattern (upregulated on day 3, peaked on day 7, normal by day 14), which showed that these two proteins are closely related to cell proliferation activation during Habu nephritis (Fig. 5b). Moreover, we also detected negative cell cycle regulatory proteins, including p53, p21 and Bax (Fig. 5c). The expression pattern of p53 was upregulated from days 3 to 14, peaking from days 7 to 14. There was no change in the expression of Bax or p21 during Habu nephritis.

## Discussion

In this study, we investigated the MC proliferative and apoptotic statuses and expression patterns of cyclins and p53 during the morbid phase of Habu nephritis. PAS staining results showed the pathological process to be consistent with previous reports [16, 17]. We examined serum creatinine and urea nitrogen levels and the urinary albumin/creatinine ratio and found inconsistent tendencies among these three indicators (the urinary albumin/creatinine ratio began to rise on day 1, whereas the other two indicators did not increase until day 3). The rise in urea albumin/creatinine ratio was closely related to glomerular filtration barrier damage during the mesangiolysis phase; this was supported by the report that snake venom induced glomerular basement membrane abnormalities, reducing the number and width of podocyte pedicels, damaging glomerular filtration barriers, and leading to large amounts of proteinuria during the mesangiolysis phase [18]. However, the upregulation of serum creatinine and blood urea nitrogen levels, appearing from days 3 to 7, were related to a significant decrease in the glomerular filtration rate at these two time points. Therefore, different indicators reflect various renal function injuries in Habu nephritis.

MC proliferation and apoptosis were found to represent crucial mechanisms for pathogenesis and recovery during Habu nephritis. In this study, we detected MC proliferation status by PCNA and KI67 staining and apoptosis by TUNEL assay. During one phase of mesangiolysis, we found that MC proliferation was activated (day 3). We deduced that the upregulation of growth factors, such as VEGF and PDGF, and inflammatory factors, such as TGF-β, may be the crucial reason for activation of MC proliferation [19], which promotes the repair process by filling the glomerular cyst with MCs. During the mesangial proliferation phase, cell proliferation, as the dominant biological process, promotes the repair of injuries caused by mesangiolysis. However, we found that the rate of apoptosis also increased. We suggest that apoptosis could suppress over-proliferation and protect renal function. During the later phase of mesangial proliferation (day 14), the PCNA and KI67 positive rate were recovered, while the apoptosis rate peaked, indicating termination of mesangial proliferation and commencement of pathological recovery induced by apoptosis. This result explored the complex MC growth changes observed in Habu nephritis.

Cell cycle regulatory proteins play a crucial role in mediating cell proliferation, and different cyclins regulate MC proliferation by different mechanisms. Cyclin D1 is a key regulatory protein of early G1 progression in MCs both in vivo and in vitro [20–22]; cyclin E is required for the G1/S transition and initiation of DNA synthesis [13]; and cyclin A2 often peaks rapidly at the onset of the S phase (or late G1), persisting through the G2 phase, which is essential for DNA synthesis and transition from the G2 to M phase [13]. In our study, we found that cyclins D1, A and E were activated during different phases, which may be related to various cytokine or protein stimuli. During the early phase of Habu nephritis, platelet and platelet secretory proteins may induce cyclin D1 synthesis [23], while during the later phase of mesangiolysis and the early phase of mesangial proliferation, activation of growth and inflammatory factors, such as VEGF, PDGF and TGF-β, may stimulate cyclins A and E [19]. Therefore, the activation of cyclins during different phases mediates the pathological process of Habu nephritis. This result may be helpful to elucidate changes in cyclins in human glomerular disease. In human crescentic glomerulonephritis, the activation of cyclin A may be crucial for cell proliferation [24], while in focal segmental glomerulosclerosis patients, cyclins E, A, and B1 and CDK2 are involved in cell proliferation [25]. These results support that different cyclin inhibitors should be applied to various types of mesangial proliferative nephritis, or even during different phases of therapy.

In addition to cell cycle regulatory proteins, the p53 signaling pathway is another important factor that promotes MC apoptosis [26–28] and inhibits cell proliferation. In IgA nephritis, the activation of p53 can promote the recovery of mesangial proliferation [29, 30]. In this study, we found that p53 was upregulated from days 3 to 14, peaking from days 7 to 14, and was involved in regulation of MC apoptosis and proliferation in Habu nephritis. There are two classical apoptotic p53 pathways: extrinsic and intrinsic. The extrinsic pathway involves engagement of specific death receptors, including Fas, DR5 and PERP, which lead to activation of caspase-8 and caspase-3, which in turn induce apoptosis [31]. The intrinsic pathway is dominated by the Bcl-2 family of proteins, such as Bax, which mediates the release of cytochrome *c* from the mitochondria [32, 33]. In this study, we found that expression of Bax, which is downstream of p53, was unchanged, which confirmed that p53 does not activate the intrinsic pathway. However, whether the extrinsic pathway was activated remains to be examined.

In conclusion, we explored MC proliferation and apoptosis and the expression patterns of cyclins and p53 during the process of Habu nephritis. This work may help elucidate the molecular pathogenesis of mesangial proliferative nephritis and improve the treatment strategy for this disease based on cell cycle control.
